# A VCO-Based CMOS Readout Circuit for Capacitive MEMS Microphones

**DOI:** 10.3390/s19194126

**Published:** 2019-09-24

**Authors:** Andres Quintero, Fernando Cardes, Carlos Perez, Cesare Buffa, Andreas Wiesbauer, Luis Hernandez

**Affiliations:** 1 Department of Electronics Technology, Carlos III University of Madrid, 28911 Leganes, Spain; fcardes@ieee.org (F.C.); caperezc@ing.uc3m.es (C.P.); luish@ing.uc3m.es (L.H.); 2 Infineon Technologies Austria AG, 9500 Villach, Austria; cesare.buffa@infineon.com (C.B.); andreas.wiesbauer@infineon.com (A.W.)

**Keywords:** MEMS microphone, oscillator-based sensor, sigma-delta modulation, time-domain circuit, VCO-ADC

## Abstract

Microelectromechanical systems (MEMS) microphone sensors have significantly improved in the past years, while the readout electronic is mainly implemented using switched-capacitor technology. The development of new battery powered “always-on” applications increasingly requires a low power consumption. In this paper, we show a new readout circuit approach which is based on a mostly digital Sigma Delta (ΣΔ) analog-to-digital converter (ADC). The operating principle of the readout circuit consists of coupling the MEMS sensor to an impedance converter that modulates the frequency of a stacked-ring oscillator—a new voltage-controlled oscillator (VCO) circuit featuring a good trade-off between phase noise and power consumption. The frequency coded signal is then sampled and converted into a noise-shaped digital sequence by a time-to-digital converter (TDC). A time-efficient design methodology has been used to optimize the sensitivity of the oscillator combined with the phase noise induced by 1/f and thermal noise. The circuit has been prototyped in a 130 nm CMOS process and directly bonded to a standard MEMS microphone. The proposed VCO-based analog-to-digital converter (VCO-ADC) has been characterized electrically and acoustically. The peak signal-to-noise and distortion ratio (SNDR) obtained from measurements is 77.9 dB-A and the dynamic range (DR) is 100 dB-A. The current consumption is 750 μA at 1.8 V and the effective area is 0.12 mm${}^{2}$. This new readout circuit may represent an enabling advance for low-cost digital MEMS microphones.

## 1. Introduction

The fast growth of the Internet of Things (IoT) and the upward trend of the mobile market are increasing the use of voice communication and speech recognition applications [1,2,3]. In some cases, an always-listening function is required to interact with the user voice commands, consequently, a low power consumption should be a key feature of such systems. In addition, low cost, small size, and easy integration are often the main imposed requirements for a microphone design. In recent years, to meet these basic needs, conventional Electret Condenser Microphones (ECM) have been replaced by Microelectromechanical systems (MEMS) sensors [4,5,6,7]. The MEMS high signal-to-noise ratio (SNR), good sensitivity, and the possibility to place a large number of these sensors in the same device make this technology well suited for current audio applications [8,9].

The low bandwidth of the audio signals enables the design of interfaces for MEMS acoustic sensors, usually employing oversampled Sigma Delta (ΣΔ) data converters [10,11]. ΣΔ modulators based on a switched-capacitor (SC) technology have a larger power consumption in comparison with continuous-time (CT) ΣΔs, but with a more robust behavior against circuit impairments like modulator clock jitter [12]. Current deep submicron CMOS technologies have led to working with reduced power supply voltages, what makes this challenging is the analog design of either SC-ΣΔs or CT-ΣΔs. However, this technology scaling impacts the performance of digital circuits in a positive way, especially in terms of area and power consumption.

Recently, time-to-digital conversion is becoming a good solution to overcome the challenges that low supply voltages pose to conventional analog circuit designs [13,14,15]. An alternative option to replace ΣΔ modulators are the oscillator-based analog-to-digital converters (ADCs). They are noise-shaped architectures wherein the information is encoded into the frequency of a digital signal; in contrast with the classical voltage encoding, therefore, the dynamic range (DR) is not limited by the power supply. In addition, they can be mostly implemented with digital circuits, taking advantage of the technology integration benefits and lower power consumption.

**Figure 1:**
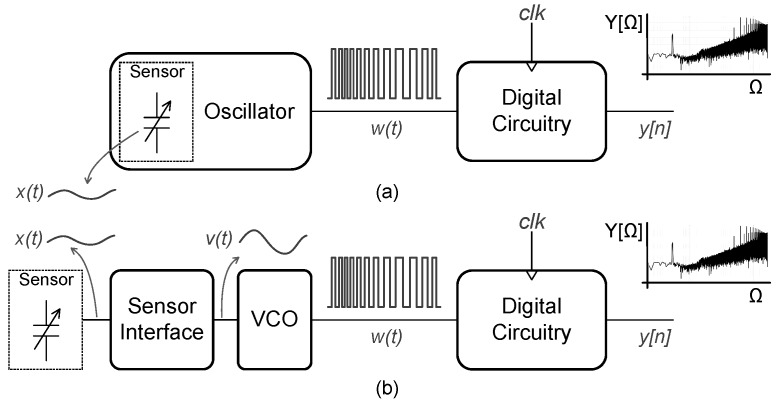
Oscillator-based analog-to-digital converter (ADC) for capacitive Microelectromechanical systems (MEMS) sensors: (**a**) Sensor connected as the load of an oscillator; (**b**) sensor connected via an analog interface.

As shown in Figure 1a, readout circuits for capacitive MEMS sensors can be done by directly connecting the sensing element as the load of an oscillator [16,17]. This way, the reactance change in the sensor modulates the oscillation frequency. By using some digital circuitry, this frequency-modulated signal can be converted into a noise-shaped bit stream. Alternatively, in Figure 1b, the sensor can be connected via an analog interface, generating a voltage signal proportional to the MEMS acoustic stimuli, which can be converted to the digital domain by a combination of a voltage-controlled oscillator (VCO) and digital circuitry. Both approaches behave like a CT-ΣΔ modulator, showing the first-order noise-shaping property at its output spectrum.

Typically, VCO-based analog-to-digital converters (VCO-ADCs) are implemented with ring oscillators (ROs) [18,19,20,21,22]. As in a conventional ADC, the VCO-ADC performance is limited by flicker and thermal noise. This circuit noise appears as phase noise in the RO, which is demodulated at the output, after sampling, as a low frequency noise affecting the overall system SNR. Another factor that limits the DR in VCO-ADC open-loop configuration is the distortion resulting from the nonlinear relationship between the input voltage and the RO oscillation frequency for large input signals. Due to these limitations, a careful design of the RO present in the ADC is required.

This paper introduces a pseudo-differential architecture for a MEMS microphone readout circuit based on a mostly digital ΣΔ ADC. The MEMS sensor is coupled to an impedance converter that modulates the frequency of a RO. The time-encoded output data of the RO is sampled with a time-to-digital converter (TDC). Finally, a thermometer-to-binary (T2B) encoder and a binary adder generate a multibit noise-shaped digital signal, which can be easily transformed into any standard audio interface by means of digital signal processing, without requiring a data-weighted averaging (DWA) technique or feedback digital-to-analog converter (DAC) linearity calibration, compared to a multibit SC-ΣΔ. Since the proposed VCO-ADC is mainly implemented with digital circuitry, it is a scalable solution in terms of area and power consumption, in contrast to SC-ΣΔ-based readout circuits for MEMS microphones. Furthermore, the SNR performance of this VCO-ADC implemented in a 130 nm CMOS process can compete with the SC-ΣΔ or the CT-ΣΔ performance [11,23].

The paper is organized as follows. Section 2 presents the system level architecture. Section 3 describes the circuit implementation at transistor level. Section 4 shows electrical and acoustical measurements obtained from the prototyped CMOS ASIC. Finally, Section 5 concludes the paper.

## 2. System Level Architecture

**Figure 2:**
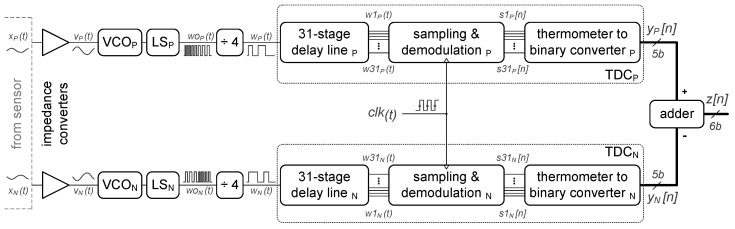
Architecture of the proposed pseudo-differential voltage-controlled oscillator (VCO)-based analog-to-digital converter (VCO-ADC).

Figure 2 shows the main blocks at the system level of the proposed VCO-ADC. It is composed of two single-ended channels which, combined in a pseudo-differential architecture, present full integration with dual-backplate (DBP) MEMS microphones. This kind of transducer is built by adding a second backplate to the conventional single-backplate (SBP) MEMS microphone, resulting in a differential capacitive sensor with even-order harmonics cancellation [8]. In the presented architecture, both the positive (P) and the negative (N) channels are identical. As a requirement of the target audio application, the output of the ADC is a multibit sequence. Nevertheless, if a single-bit signal is preferred instead, the multibit output can be processed with a noise-shaper coder.

The output of the capacitive MEMS sensor x(t) is coupled into the ADC input via an impedance converter, generating the signal v(t), which sets the frequency of the VCO. The oscillator output, after a level shifter, is divided by a factor of four in order to adjust the oscillation frequency and make it compatible with the implemented demodulation circuit. The frequency-modulated signal w(t) obtained at the output of the divider is passed through a 31-stage delay line, producing a delayed copy of w(t) for each tap. The output signals of the delay chain w1(t)–w31(t) are sampled and demodulated, applying the first-order difference ($1-z^{-1}$). By using a T2B converter, the demodulated signals s1[n]–s31[n] form the multibit signal y[n]. The digital output of the converter z[n] is given by the two’s-complement subtraction of both single-ended branches.

The signal level applied to the microphone is expressed in a logarithmic scale, assuming 0 dB${}_{\mathrm{SPL}}$ as a reference for the human hearing threshold of 20 μPa of sound pressure level (SPL). The sensitivity of the DBP MEMS to be used in the proposed ADC is 12 mV${}_{\mathrm{rms}}/\mathrm{Pa}$ (94 dB${}_{\mathrm{SPL}}$).

As mentioned above, the voltage signal v(t) controls the frequency of wo(t), being the frequency variation in the VCO proportional to the magnitude sensed by the transducer connected to the ADC. Due to power consumption and noise reasons, it would be advisable that the target frequency of signal w(t) in Figure 1 is $f_{0}$ = 4 MHz and the oscillator gain should be $k_{\mathrm{vco}}$ = 12 MHz/V, with a relative frequency deviation $k_{d}$ = $k_{\mathrm{vco}}$/$f_{0}$ = 3 V${}^{-1}$. Given that the instantaneous oscillation frequency is

(1) $$f\left(t\right)=f_{0}\cdot \left(1+k_{d}\cdot v\left(t\right)\right),$$

these VCO design parameters set a limit for the single-ended peak amplitude of v(t) close to ±333 mV (≈126 dB${}_{\mathrm{SPL}}$), but this is only an ideal assumption. Actually, certain factors like the distortion components can be seen as a frequency variation caused by the input signal.

Currently use of low order modulators is due to the development of new audio interfaces like MIPI SoundWire^®^. It includes support for multiple data rates in the order of tens of megahertz, which is higher than the standard sampling rates of ΣΔ ADCs, reducing the need for high-order modulators. In this design, the sampling frequency ($f_{s}$) is 20 MHz. However, depending on the VCO quantization architecture, this sampling rate might not be enough to achieve the resolution required for audio applications.

For example, using a single reset counter that counts the edges of w(t) in a sampling clock period, the theoretical signal-to-quantization-noise ratio (SQNR) that could be achieved in Figure 1 is

(2) $$SQNR=6.02\cdot log_{2}\left(\frac{2AK_{VCO}}{f_{s}}\right)-3.41+30\cdot log_{10}OSR+20\cdot log_{10}\left(sinc\left(\frac{1}{2OSR}\right)\right),$$

where *A* is the amplitude of the input signal and OSR is the oversampling ratio equals $f_{s}$/(2BW) [19]. Applying Equation (2) and assuming a differential input signal with *A* = 16.97 mV, which corresponds to 1 Pa (94 dB${}_{\mathrm{SPL}}$) of sound pressure in the target MEMS microphone, with the VCO parameters depicted before, the estimated SQNR of this configuration will be limited to 47.74 dB over the audio bandwidth (BW = 20 kHz). Instead, a valid alternative is the use of a TDC for the quantization of the time-encoded signal w(t), which emulates a much higher sampling rate than the actual sampling clock frequency. By using this solution, we can get a multibit sequence presenting an enhanced SQNR without increasing the system clock frequency and with an excellent trade-off between the VCO oscillation frequency, the number of VCO phases, and the number of stages in the TDC.

**Figure 3:**
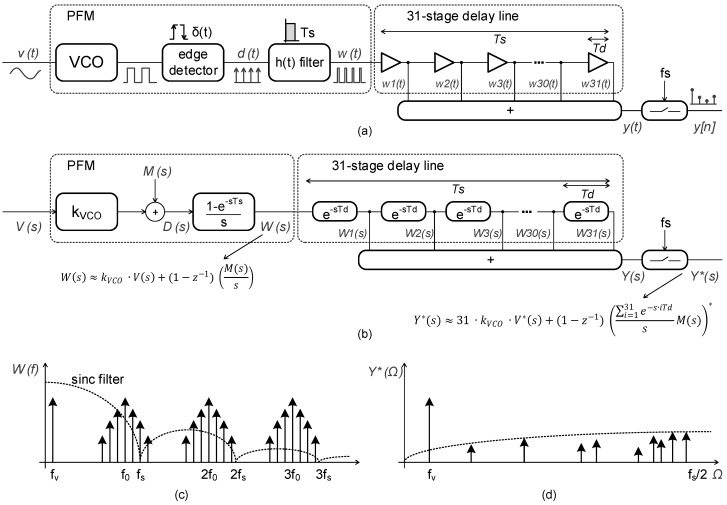
Pulse frequency modulation VCO-ADC: (**a**) Time model; (**b**) Laplace model; (**c**) filtered signal w(t); (**d**) sampled output y[n].

In this paper, we propose a different approach based on a high-rate sampler that interpolates samples between two clock edges and implements an analog finite-impulse-response (FIR) decimator. This system can be better explained using the pulse frequency modulation (PFM) approach introduced in [24,25]. In Figure 3a, signal v(t) is passed through a PFM modulator composed of a VCO, an edge detector block, and a pulse-shaping filter h(t). According to [25], the PFM modulator behaves like a signal coder, whose output spectrum reproduces the input signal together with some modulation components M(s) (Figure 3c). For band-limited signals, M(s) lie at frequencies much higher than the input signal.

As a virtue of the pulse shaping filter h(t), the spectrum of signal w(t) also has nulls at the multiples of $f_{s}$ (Figure 3c). These nulls provide a first-order spectral shaping after sampling of components M(s) and their aliases (Figure 3d). To further improve the SQNR, we add a low pass FIR filter implemented with continuous time delays, as shown in Figure 3b. The FIR filter reduces the level of modulation components M(s) prior to sampling. As a consequence, the signal at the output of the PFM modulator can be converted into a multibit signal. As an intuitive explanation of the FIR filter operation, the power of the input signal is now multiplied by the number of stages of the delay line, while the modulation components are filtered by this extra low-pass FIR filter, as evidenced in Figure 3b. This results in an ADC resolution enhancement.

**Figure 4:**
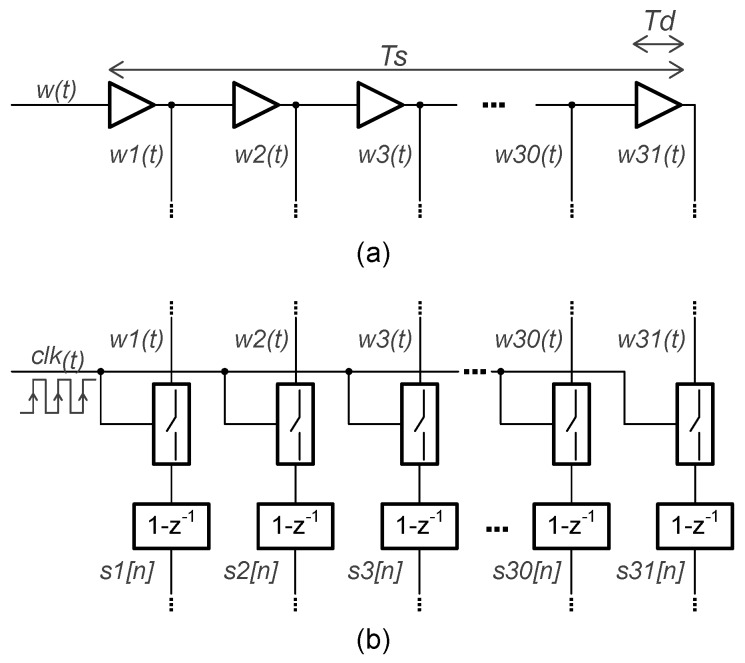
Time-to-digital converter: (**a**) 31-stage delay line; (**b**) sampling and demodulation schema.

The first component of the TDC is the 31-stage delay line, shown in Figure 4a. The ideal delay time of the entire chain corresponds to the sampling period of the system, denoted by $T_{s}$ = $1/f_{s}$. Ideally, the time delay of every single element is $T_{d}$ and equals $T_{s}$/31. By implementing this number of basic delay units, the resolution achieved in the proposed first-order VCO-ADC will be enough for an audio application, as will be shown afterwards from behavioral simulations. Also, the required value of $T_{d}$ in every delay unit can be implemented with the selected CMOS process employing basic digital buffers.

The output taps of the delay line w1(t)–w31(t) are registered with the system clock and the first-order difference is applied, as shown in Figure 4b. Then, using the T2B encoder, the demodulated single-bit sequences s1[n]–s31[n] are combined into a 5-bit signal y[n]. This is the output of the single-ended channel, and it shows a first-order noise-shaping property at its power spectrum. The pseudo-differential output of the VCO-ADC is the 6-bit sequence z[n] which, unlike the single-ended configuration, cancels the even harmonic distortion components.

**Figure 5:**
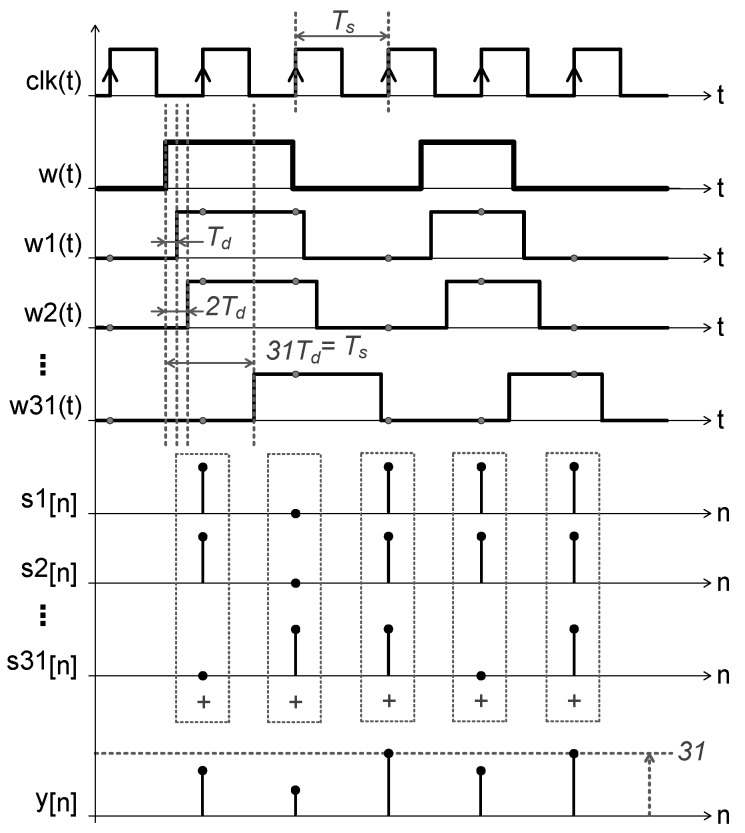
Chronogram of digital signals in the single-ended channel configuration.

Figure 5 depicts the behavior of the quantization method applied in both the single-ended channels. The signal w(t) is delayed by a time $T_{d}$ in each of the delay elements of the TDC, w31(t) being the last delayed component with a delay time equivalent to $T_{s}$. The states of the signals w1(t)–w31(t) are sampled at the rising edges of the clk signal. The first difference of these sampled data is computed, generating the discrete sequences s1[n]–s31[n]. Finally, the multibit signal y[n] is given by the sum of the values of these discrete sequences at every sampling period.

**Figure 6:**
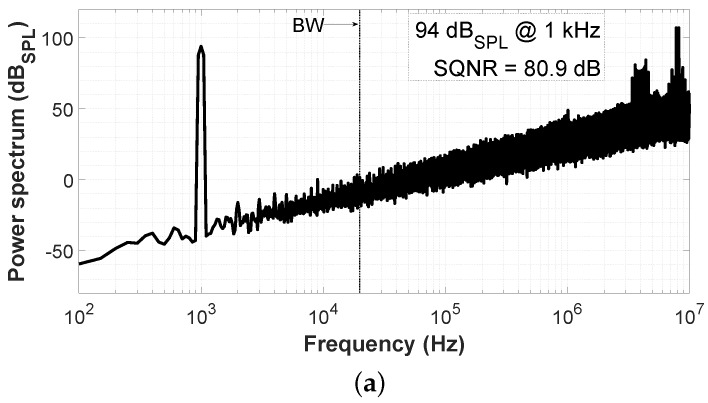

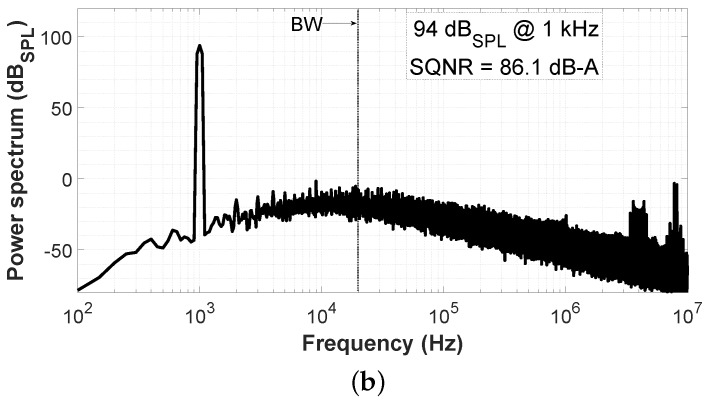
Simulated spectrum for an input signal of 94 dB${}_{\mathrm{SPL}}$ at 1 kHz: (**a**) Non-A-Weighted; (**b**) A-Weighted.

Figure 6 shows the result of a behavioral simulation of the VCO-ADC architecture presented in Figure 2 without the impedance converters. The input-referred spectra have been calculated using an input tone corresponding to 94 dB${}_{\mathrm{SPL}}$ at 1 kHz. The SQNR obtained in the audio bandwidth under these conditions is 80.9 dB and 86.1 dB-A if an A-Weighting filter is applied (Figure 6b). Note that the simulated spectra show first-order noise shaping. The A-Weighting curve is commonly used in audio measurements to mimic the sound pressure detected by the human ear, which is less sensitive to low audio frequencies.

### Analysis of Nonidealities

As mentioned in Section 1, phase noise and distortion may affect the performance of the VCO implementation. The effect of phase noise in oscillators has been extensively studied in [26,27,28], concluding that phase noise is influenced by certain factors such as the topology of the oscillator, the oscillation frequency, the size of the transistors, and the power consumption. In this ADC, the VCO is optimized in terms of phase noise and distortion by applying the method described in [29]. Furthermore, the distortion is mitigated by the pseudo-differential configuration.

**Figure 7:**
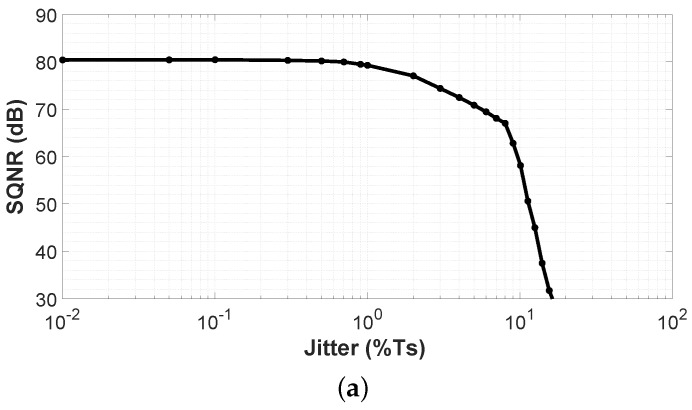

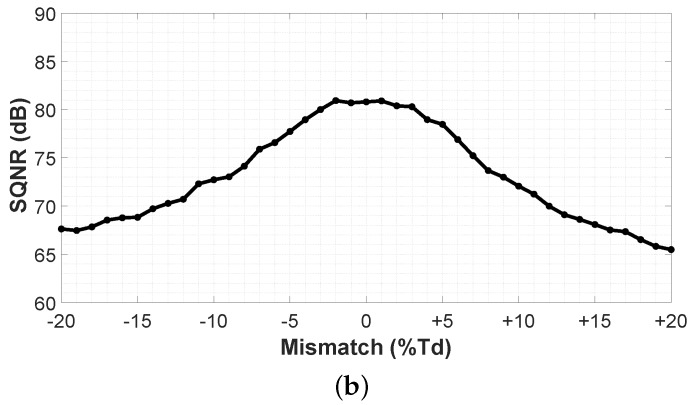
Simulated signal-to-quantization-noise ratio (SQNR) for an input signal of 94 dB${}_{\mathrm{SPL}}$ at 1 kHz with (**a**) clock jitter variation and (**b**) digital delay line mismatch.

However, jitter present in the sampling clock may have a negative impact on the performance of the proposed VCO-ADC. Jitter can be seen as a deviation of the sampling period from the ideal value and, in real implementations, its presence may be unavoidable [30]. Some approaches for the jitter sensitivity reduction in CT-ΣΔs have been published, for example in [31,32]. Figure 7a shows the simulated SQNR for the proposed system assuming different values of clock jitter. The SQNR remains without important changes up to 1% of period jitter rms value ($T_{s}$ = 50 ns). The performance of the system will be significantly degraded if a sampling clock with a jitter σ above 1% is applied.

Another possible nonideality that may adversely impact the performance of the system is the mismatch of the digital delay line. In a CMOS prototype implementation, due to Process-Voltage-Temperature (PVT) variations, the real delay time ($T_{d}$) of the delay elements could be different from the nominal case. Figure 7b shows the simulated SQNR for different values of delay mismatch, represented as a percent of $T_{d}$. A loss of 2 dB in the SQNR can be observed within a margin of ±5% of mismatch in every element of the delay line, which is a permissible variation according to the specifications posed by the target audio application.

## 3. Circuit Design

**Figure 8:**
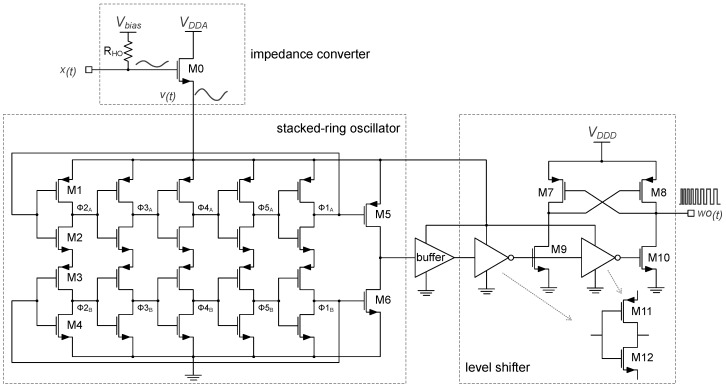
Single-ended analog core of the proposed VCO-ADC: Impedance converter, 5-stage stacked-RO, and differential cascade voltage switch level shifter.

Figure 8 shows the simplified schematic of the analog core for the single-ended channel configuration in the proposed VCO-ADC. The MEMS sensor is biased by a high-ohmic biasing circuit [8] and a NMOS (M0) transistor in the common-drain amplifier configuration. M0 acts as a voltage buffer stage that adapts the high-impedance input signal x(t) into the low-impedance output signal v(t). The DC operating point of the buffer is established by the gigaohms order bias resistor, denoted as $R_{\mathrm{HO}}$, which is implemented by two asymmetric branches of stacked PMOS diodes. The dimensions of M0 have been estimated in order to minimize its noise contribution to the overall ADC SNR and also to keep its gain close to unity.

The low phase noise, the ease of implementation, and the good sensitivity make the ROs excellent candidates to be used in VCO-ADCs [18,19,20,21,22]. In addition, one of the most important advantages of ROs is the possibility to use their multiphase output. This allows a multibit quantization approach presenting an enhanced SQNR, but at the cost of involving more complex digital circuits. In this VCO-ADC a single RO output phase has been connected to the 31-stage delay line in order to minimize the area and complexity of the digital circuitry. However, by using the implemented TDC solution, a sufficient SQNR is achieved in the 20 kHz audio bandwidth, as has already been proven by behavioral simulations in the previous Section.

The oscillator implemented in this converter is a 5-stage inverter-based RO built with two stacked rings, which shows a better phase noise compared to the conventional single-ring architecture [33]. In Figure 8, both stacked rings, connected through the NMOS devices of the upper chain of inverters to the PMOS transistors of the lower side, oscillate like a conventional RO having the same $f_{0}$, but with a difference in phase of 180${}^{\circ }$. Given that only one RO output phase has to be connected to the TDC, the stacked signals at $\phi 1_{B}$ and $\phi 1_{A}$ with amplitude *VSS*–v(t)/2 and v(t)/2–v(t), respectively, are combined into a single signal of amplitude *VSS*–v(t) by means of M5 and M6. A buffer is employed to square the RO output oscillation. The signal amplitude after the buffer is still variable and depends on the level of v(t). Therefore, the level shifter of Figure 8 is needed to keep constant logic levels at the input of the digital circuitry, regardless of the VCO-ADC input signal. This way, the level of the time-encoded signal wo(t) is always compatible with the digital supply voltage, which is $V_{DDD}$ = 1.8 V in this case. The PMOS transistors of the level shifter (M7–M8) are W/L = 1 μm/400 nm and the NMOS (M9–M10) are W/L = 4 μm/400 nm, whereas the transistors of the inverters (M11 and M12) are W/L = 1.1 μm/400 nm and W/L = 740 nm/400 nm, respectively.

The RO design process involved the methodology proposed in [29] to find an optimized oscillator in terms of phase noise, sensitivity, and distortion that benefits from a reduced simulation time. This methodology is based on periodic steady-state (*PSS*) sweep simulations to estimate the $f_{0}$, the gain, and the distortion of the RO. Additionally, the periodic noise (*pnoise*) analysis is used to compute the phase noise, which can be referred to the ADC input to predict the SNR. As shown in [29], such analyses achieve very good accuracy with an important speed up in the simulation time, which allows an interactive optimization of the RO design instead of using conventional slow transient simulations.

**Figure 9:**
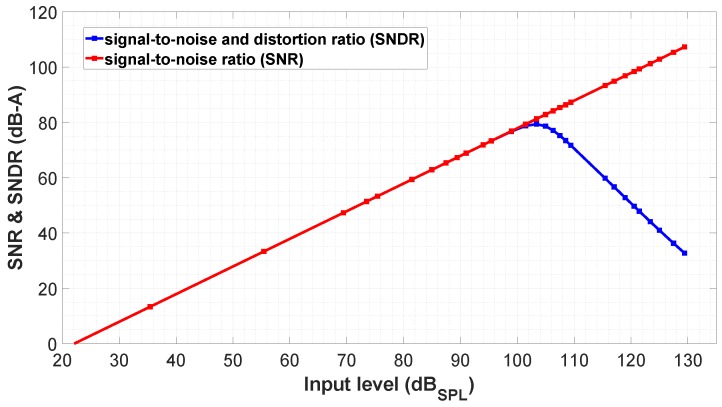
Simulated analog core dynamic range in the differential configuration for different input levels referred to 94 dB${}_{\mathrm{SPL}}$ = 12 mV${}_{\mathrm{rms}}$ (A-Weighting filter applied).

After applying this optimization process, the selected RO have W/L = 288 μm/900 nm for M1–M3 and W/L = 288 μm/1.9 μm for M2–M4 devices. This RO achieves $f_{0}$ = 16.2 MHz and $k_{d}$ = 3.04 V${}^{-1}$, which are very close to the target values mentioned in Section 2. Figure 9 shows a predicted DR for the differential oscillator configuration, which has been estimated from the equations presented in [29]. To get these estimations, the RO simulation setup included the noise contribution of the impedance converter supplied at $V_{DDA}$ = $V_{bias}$ = 1.8 V. The peak signal-to-noise and distortion ratio (SNDR) predicted is 80 dB-A. Note that the TDC quantization noise is not considered here and also has an impact on the overall VCO-ADC SNDR, as will be shown in Section 4.

**Figure 10:**
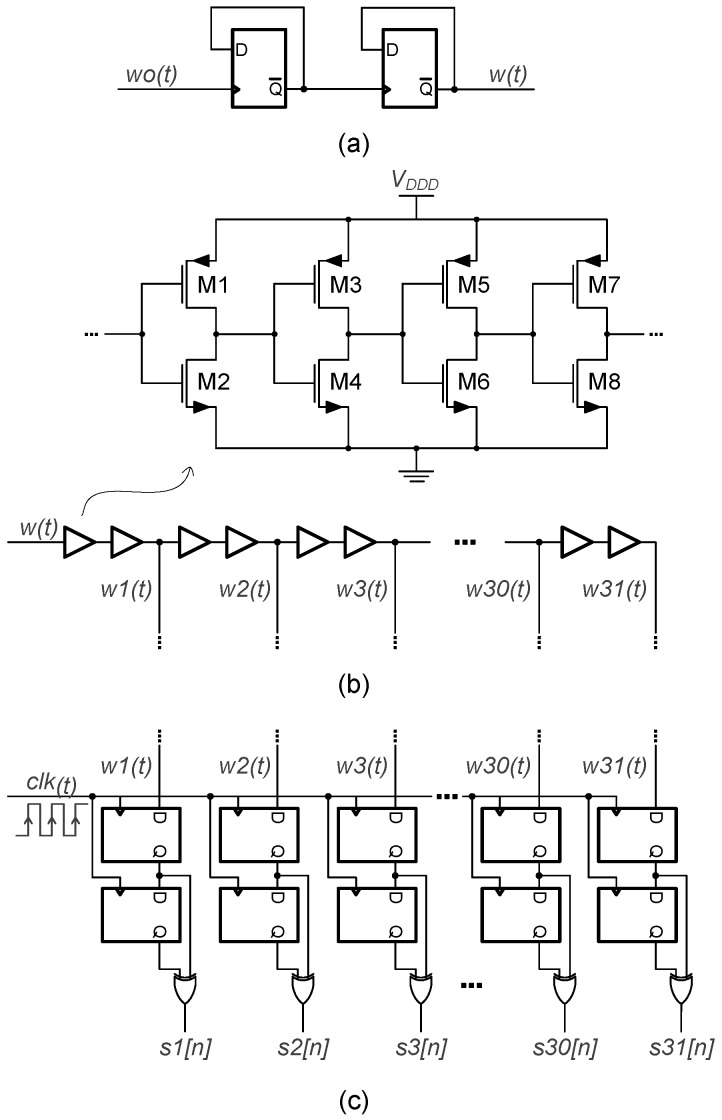
Single-ended digital circuitry of the proposed VCO-ADC: (**a**) Frequency divider; (**b**) 31-stage delay line; (**c**) sampling and demodulation circuit.

Figure 10 illustrates some of the digital blocks of the proposed VCO-ADC. The frequency division of the RO output is performed by the two D-type Flip-Flops of Figure 10a. Here, the inverted output terminal of each Flip-Flop is connected to the data input terminal, resulting in a division by a factor of four of the first Flip-Flop clock signal. The composition of the 31-stage delay line is shown in Figure 10b. Every delay unit is formed by two digital buffers, each one implemented with four inverters, where W/L(M1) = 750 nm/400 nm, W/L(M2) = 500 nm/400 nm, W/L(M3–M5) = 500 nm/500 nm, W/L(M4–M6) = 500 nm/1 μm, W/L(M7) = 1.42 μm/400 nm, and W/L(M8) = 920 nm/400 nm. Post-layout simulations show a total delay time of the entire chain equal to 47 ns, which is very close to the specified ADC sampling period of 50 ns. This leads to an individual delay time $T_{d}$ = 1.52 ns in each unit. It is important to note that to achieve a total delay time closer to $T_{s}$ requires a big effort, since the delay time of each unit is highly dependent on its layout implementation. Monte Carlo simulation results show that the $T_{d}$ standard deviation value is within 5% of the margin, so the delay cell mismatch does not cause a negative impact on the system performance, as proven in Section 2.

In the proposed VCO-ADC, the specified $f_{s}$ is higher than twice the maximum oscillation frequency after the divider. This allows the use of the XOR-based demodulation circuit of Figure 10c to compute the first-order difference for the delayed copies of the RO output [19,34]. This circuit accounts for the oscillation rising and falling edges, as already described in Figure 5. The remaining blocks of the digital core that process the demodulated single-bit sequences s1[n]–s31[n] are the T2B and the differential two’s-complement subtractor. Both of them are implemented with an array of full adders to generate the 6-bit ADC output, without carry propagate functions.

## 4. Experimental Results

**Figure 11:**
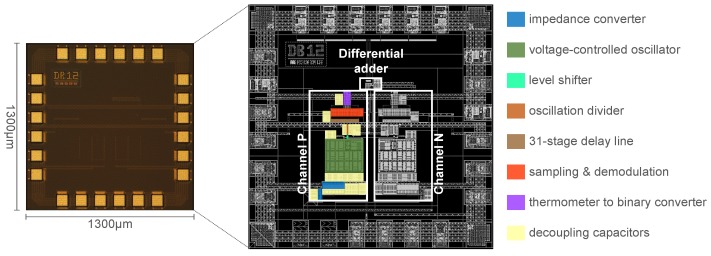
Die micrograph and area distribution.

The proposed VCO-ADC has been fabricated in a 130 nm standard CMOS process. Figure 11 shows the die dimensions, occupying a total area of 1.69 mm${}^{2}$. As can be observed from the block distribution, only 7% of the die area is used by the ADC components, resulting in an active area of 0.12 mm${}^{2}$. In the layout, both ROs have been well spaced to avoid the injection-locking effect between them. In addition, for this purpose, the ROs have been protected using guards rings. The ASIC pads include the MEMS microphone interface, the digital data output, the voltage supplies, the clock input, and some test signals like the ROs output.

### 4.1. Electrical Measurements

To evaluate the performance of the proposed VCO-ADC architecture, the implemented ASIC has been packaged together with a silicon capacitor array directly bonded to the ADC input, in order to emulate the real capacitance of a DBP MEMS microphone. Then, the differential ADC input signal is injected through these capacitors using the balanced output of an audio function generator. This way, by applying the proper input signal levels, the ADC behaves as if the MEMS microphone would be connected to the converter input. The 1.8 V for the $V_{bias}$, $V_{DDA}$, and $V_{DDD}$ have been generated with batteries and using discrete LDO regulators to avoid the impact of the noise present in switching power supplies. To minimize the clock jitter, a commercial crystal oscillator of 20 MHz was employed as the system clk. The 6-bit VCO-ADC digital output was acquired using a logic analyzer. All the data postprocessing was done on a PC using MATLAB^®^.

**Figure 12:**
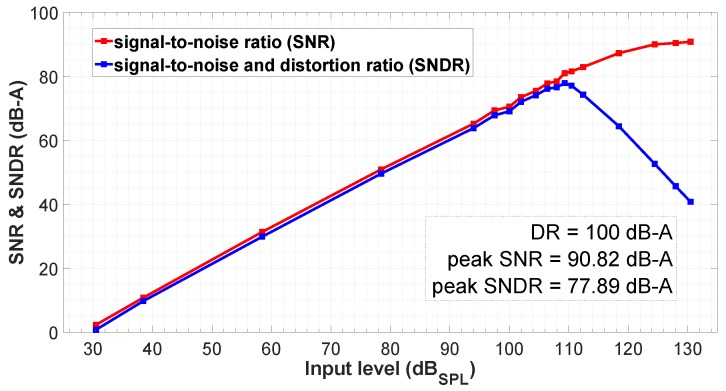
Measured dynamic range for different input levels referred to 94 dB${}_{\mathrm{SPL}}$ = 12 mV${}_{\mathrm{rms}}$ (A-Weighting filter applied).

Figure 12 shows the measured DR for a level amplitude sweep of a differential input tone at 1 kHz and applying the A-Weighting filter, as usual in audio applications. In this case, the DR reaches 100 dB-A, with a peak SNR equal to 90.82 dB-A and a peak SNDR of 77.89 dB-A. The acoustic overload point (AOP) (when the maximum allowed distortion is reached) is at 130 dB${}_{\mathrm{SPL}}$. The obtained AOP for a SNDR = 40 dB-A is good enough for low-cost digital microphones [20]. This translates into a reduced complexity of the proposed architecture since there is no need for linearity compensation techniques, which may have a negative impact on area and power consumption [22].

**Figure 13:**
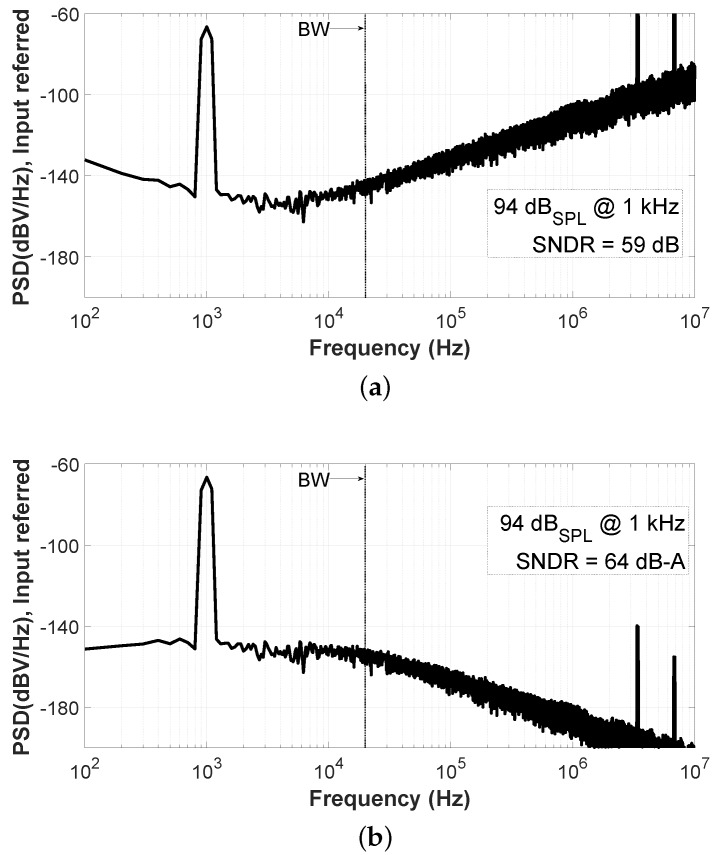
Measured spectrum for an input signal of 94 dB${}_{\mathrm{SPL}}$ (12 mV${}_{\mathrm{rms}}$) at 1 kHz: (**a**) Non-A-Weighted; (**b**) A-Weighted.

The spectra of Figure 13 are obtained applying in the function generator the signal equivalent to 1 Pa of sound pressure in the MEMS microphone, that is, the 94 dB${}_{\mathrm{SPL}}$ reference at 1 kHz. The achieved SNDR is 59 dB or 64 dB-A if the A-Weighting filter is applied (Figure 13b). At this level, the SNDR limit is imposed only by the flicker, thermal, and quantization noise. Note that the flicker noise is attenuated by the A-Weighting curve. The sidebands of the VCO appear as high-frequency components.

**Figure 14:**
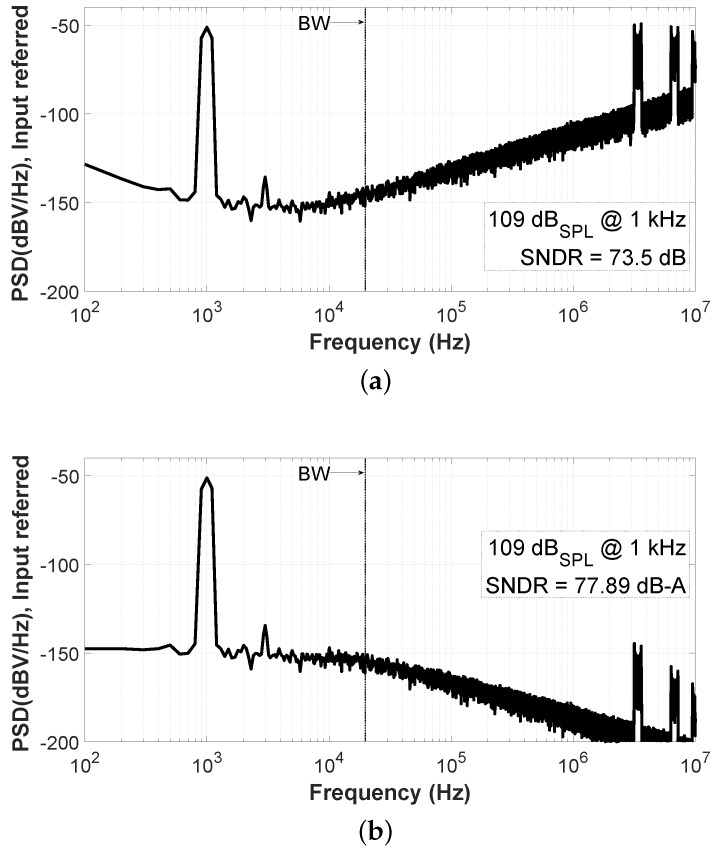
Measured spectrum for an input signal of 109 dB${}_{\mathrm{SPL}}$ (67.5 mV${}_{\mathrm{rms}}$) at 1 kHz: (**a**) Non-A-Weighted; (**b**) A-Weighted.

Another interesting point corresponds to the level for the maximum SNDR. This condition happens when the acoustic input level reaches 109 dB${}_{\mathrm{SPL}}$ in the sensor. Figure 14 shows the ADC output spectra when the SNDR is maximized. It can be seen that, due to the pseudo-differential configuration, the 2nd harmonic component is canceled but the 3rd one starts to be visible. The distortion for higher input levels is limited by the nonlinearity of the ROs. Moreover, different input signal levels produce wider or narrower modulation sidebands between spectra in Figure 13 and Figure 14, as shown in [24].

**Figure 15:**
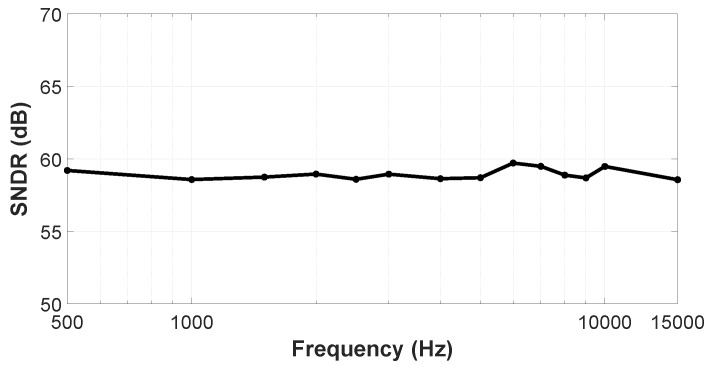
Measured frequency response (500 Hz to 15 kHz) for an input signal of 94 dB${}_{\mathrm{SPL}}$ (12 mV${}_{\mathrm{rms}}$).

Figure 15 shows the VCO-ADC frequency response from 500 Hz to 15 kHz for a 94 dB${}_{\mathrm{SPL}}$ equivalent input. In this range, the SNDR shows a maximum variation close to 1 dB. Note that the A-Weighting filter is not applied here, in order to avoid the nonflat characteristic of the A-Weighting curve in the measured frequency response.

### 4.2. Acoustical Measurements

**Figure 16:**
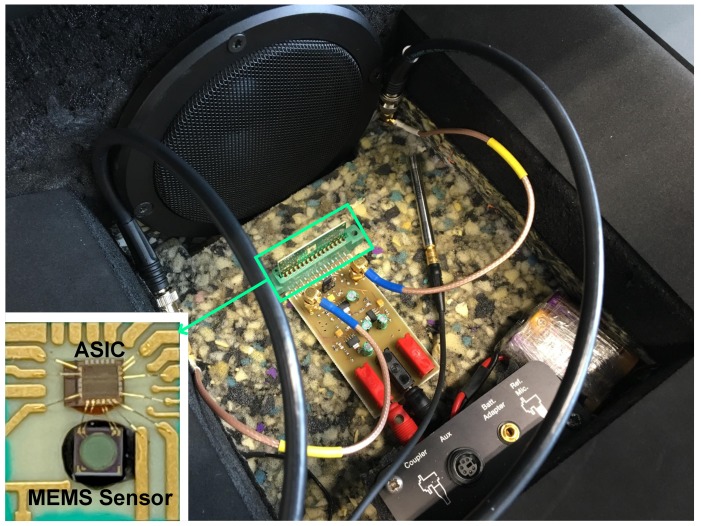
Test fixture used in the acoustical measurements.

Apart from the described electrical evaluations, some acoustical measurements have been performed. This time, the implemented ASIC and the DBP MEMS microphone have been directly bonded over a low-leakage PCB, which includes a sound port in the sensor bottom, enabling the contact of sound pressure against the MEMS membrane. To exclude the external acoustic noise and to keep the level of the audio signal applied during the measurements under control, an anechoic test box has been used. A box built-in speaker was employed to generate the audio test tone. Given that the TDC performance was checked in the electrical measurements, in order to keep the simplicity of the test PCB and to reduce the wire connections out of the box, only the ROs test output was acquired with an oscilloscope and then postprocessed in MATLAB^®^. Figure 16 depicts the test fixture used for the acoustical characterization of the sensor.

**Figure 17:**
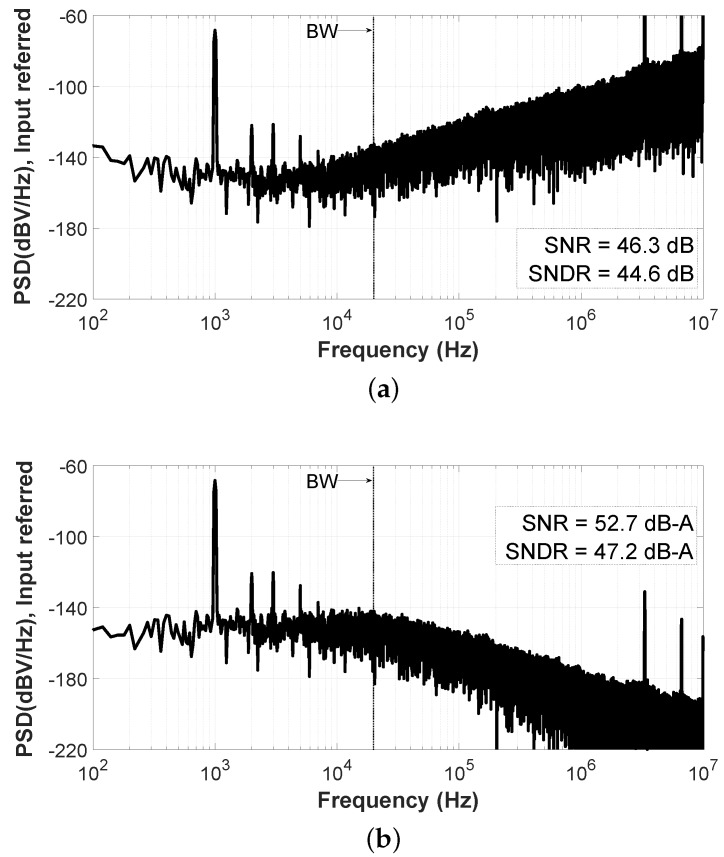
Measured spectrum for an acoustic tone of 94 dB${}_{\mathrm{SPL}}$ at 1 kHz: (**a**) Non-A-Weighted; (**b**) A-Weighted.

Figure 17 shows the differential ROs output spectra for an audio tone of 94 dB${}_{\mathrm{SPL}}$ at 1 kHz, achieving a SNR = 46.3 dB and the SNDR = 44.6 dB (Figure 17a). With the A-Weighting correction, the SNR reaches 52.7 dB-A and the SNDR equals 47.2 dB-A (Figure 17b). The difference in sensitivity and distortion with the results presented in Figure 13 is due to two main facts. First, in the test fixture shown in Figure 16, a MEMS acoustic package is missing, so the back volume of the MEMS microphone becomes infinite. The optimum mechanical sensitivity of the MEMS is achieved for a specific back volume, which is accurately defined in the packaging process. Moreover, to obtain a good sensitivity, the MEMS membrane should be biased with a high-voltage signal in the order of 6–9 V, depending on the MEMS characteristics. Usually this is achieved with an on-chip charge pump, which has not been included in this test chip. Anyhow, the most important outcome from the acoustical measurements is that the proposed VCO-ADC architecture is audio-responsive, presenting fully compatibility with the MEMS microphone sensors.

Measurements show that the implemented VCO-ADC architecture has a current consumption of 750 μA at 1.8 V, including all the blocks of Figure 11. The Schreier figure-of-merit (FoM), expressed as FoM${}_{S}$ = DR + 10log${}_{10}$ (BW/Power), is 171.7 dB.

The measurement results presented in this section are valid for the case when a sampling clock with a reduced jitter is employed. As shown in Section 2, the proposed VCO-ADC shows a good sensitivity against clock jitter but SC-ΣΔs are less tolerant to this problem. Another disadvantage of the proposed oscillator-based architecture is the possible injection-locking between ROs. Some actions should be taken in the layout implementation to avoid this effect.

## 5. Conclusions

This paper presents a pseudo-differential VCO-based readout circuit for a capacitive MEMS sensor, suitable for low-cost digital microphones. The proposed VCO-ADC architecture is mostly digital, being a CMOS scalable alternative solution in terms of area and power consumption, in contrast to SC-ΣΔ-based readout circuits for MEMS microphones. It is mainly composed of a stacked-ring oscillator and a time-to-digital converter. A prototype has been fabricated in 130 nm CMOS process to validate the proposed circuit by means of electrical and acoustical measurements, occupying an effective area of 0.12 mm${}^{2}$. Electrical measurements exhibit a peak SNR of 90.8 dB-A and a peak SNDR of 77.9 dB-A. The dynamic range reached is 100 dB-A and the acoustic overload point is 130 dB${}_{\mathrm{SPL}}$, with a current consumption of 750 μA powered at 1.8 V. Finally, acoustical measurements using a DBP MEMS microphone, show that the proposed ADC architecture is a good candidate to replace the traditional ΣΔ-based readout circuits in audio applications. A future line of research may concentrate on improving the power consumption of the digital architecture by extending the time-to-digital converter to all the VCO phases or implementing a higher-than-sampling frequency VCO coupled to some phase-counting circuit.

## References

[1] Kim Y., Oh H., Kang S. (2017). Proof of Concept of Home IoT Connected Vehicles. Sensors.

[2] Gil D., Ferrández A., Mora-Mora H., Peral J. (2016). Internet of things: A review of surveys based on context aware intelligent services. Sensors.

[3] Ghayvat H., Mukhopadhyay S., Gui X., Suryadevara N. (2015). WSN-and IOT-based smart homes and their extension to smart buildings. Sensors.

[4] Peña-García N., Aguilera-Cortés L., González-Palacios M., Raskin J.P., Herrera-May A. (2018). Design and modeling of a MEMS dual-backplate capacitive microphone with spring-supported diaphragm for mobile device applications. Sensors.

[5] Leinenbach C., van Teeffelen K., Laermer F., Seidel H. A New Capacitive Type MEMS Microphone. Proceedings of the 2010 IEEE 23rd International Conference on Micro Electro Mechanical Systems (MEMS).

[6] Neumann J.J., Gabriel K.J. A Fully-integrated CMOS-MEMS Audio Microphone. Proceedings of the 12th International Conference on Solid-State Sensors, Actuators and Microsystems.

[7] Weigold J.W., Brosnihan T.J., Bergeron J., Zhang X. A MEMS Condenser Microphone for Consumer Applications. Proceedings of the 19th IEEE International Conference Micro Electro Mechanical Systems.

[8] Bach E., Gaggl R., Sant L., Buffa C., Stojanovic S., Straeussnigg D., Wiesbauer A. 9.5 A 1.8V True-Differential 140dB SPL Full-Scale Standard CMOS MEMS Digital Microphone Exhibiting 67dB SNR. Proceedings of the IEEE International Solid-State Circuits Conference (ISSCC).

[9] Zwyssig E., Lincoln M., Renals S. A Digital Microphone Array for Distant Speech Recognition. Proceedings of the Speech and Signal Processing 2010 IEEE International Conference Acoustics.

[10] Berti C.D., Malcovati P., Crespi L., Baschirotto A. A Low-Power, Continuous-Time Sigma-Delta Modulator for MEMS Microphones. Proceedings of the 9th Conference Ph.D. Research in Microelectronics and Electronics (PRIME).

[11] De Berti C., Malcovati P., Crespi L., Baschirotto A. (2016). A 106 dB A-Weighted DR Low-Power Continuous-Time ΣΔ Modulator for MEMS Microphones. IEEE J. Solid-State Circuits.

[12] Nandi T., Boominathan K., Pavan S. (2013). Continuous-Time ΔΣ Modulators With Improved Linearity and Reduced Clock Jitter Sensitivity Using the Switched-Capacitor Return-to-Zero DAC. IEEE J. Solid-State Circuits.

[13] Watanabe T., Mizuno T., Makino Y. (2003). An all-digital analog-to-digital converter with 12-μV/LSB using moving-average filtering. IEEE J. Solid-State Circuits.

[14] Watanabe T., Terasawa T. An All-Digital ADC/TDC for Sensor Interface with TAD Architecture in 0.18-μm Digital CMOS. Proceedings of the 2009 16th IEEE International Conference on Electronics, Circuits and Systems—(ICECS 2009).

[15] Lee M., Abidi A.A. (2008). A 9 b, 1.25 ps Resolution Coarse –Fine Time-to-Digital Converter in 90 nm CMOS that Amplifies a Time Residue. IEEE J. Solid-State Circuits.

[16] Dai C.L., Lu P.W., Chang C., Liu C.Y. (2009). Capacitive micro pressure sensor integrated with a ring oscillator circuit on chip. Sensors.

[17] Soell S., Porr B. An Undersampling Digital Microphone. Proceedings of the 2007 IEEE International Symposium on Circuits and Systems (ISCAS).

[18] Daniels J., Dehaene W., Steyaert M., Wiesbauer A. A 0.02 mm^2^ 65 nm CMOS 30 MHz BW All-Digital Differential VCO-Based ADC with 64dB SNDR. Proceedings of the 2010 Symposium on VLSI Circuits.

[19] Kim J., Jang T.K., Yoon Y.G., Cho S. (2010). Analysis and design of voltage-controlled oscillator based analog-to-digital converter. IEEE Trans. Circuits Syst. I Regul. Pap..

[20] Cardes F., Gutierrez E., Quintero A., Buffa C., Wiesbauer A., Hernandez L. (2018). 0.04-mm^2^103-dB-A Dynamic Range Second-Order VCO-Based AudioΣΔADC in 0.13-*μ*m CMOS. IEEE J. Solid-State Circuits.

[21] Danesh M., Chandrasekaran S.T., Sanyal A. Ring Oscillator Based Delta-Sigma ADCs. Proceedings of the 2018 25th IEEE International Conference on Electronics, Circuits and Systems (ICECS).

[22] Alvero-Gonzalez L.M., Gutierrez E., Hernandez L. A Highly Linear Ring Oscillator for VCO-based ADCs in 65-nm CMOS. Proceedings of the Circuits and Systems (ICECS) 2018 25th IEEE International Conference Electronics.

[23] Billa S., Sukumaran A., Pavan S. A 280*μ*W 24kHz-BW 98.5 dB-SNDR Chopped Single-bit CT ΔΣM Achieving< 10Hz 1/f Noise Corner without Chopping Artifacts. Proceedings of the ISSCC.

[24] Gutierrez E., Hernandez L., Cardes F., Rombouts P. (2018). A pulse frequency modulation interpretation of VCOs enabling VCO-ADC architectures with extended noise shaping. IEEE Trans. Circuits Syst. I Regul. Pap..

[25] Gutierrez E., Perez C., Hernandez L., Cardes F., Petrescu V., Walter S., Gaier U. (2019). A pulse frequency modulation VCO-ADC in 40 nm. IEEE Trans. Circuits Syst. II Express Briefs.

[26] Razavi B. (1996). A study of phase noise in CMOS oscillators. IEEE J. Solid-State Circuits.

[27] Hajimiri A., Limotyrakis S., Lee T.H. (1999). Jitter and phase noise in ring oscillators. IEEE J. Solid-State Circuits.

[28] Abidi A.A. (2006). Phase Noise and Jitter in CMOS Ring Oscillators. IEEE J. Solid-State Circuits.

[29] Cardes F., Quintero A., Gutierrez E., Buffa C., Wiesbauer A., Hernandez L. (2018). SNDR Limits of Oscillator-Based Sensor Readout Circuits. Sensors.

[30] Cardes F., Medina V., Paton S., Hernandez L. (2019). Clock Jitter Analysis of Continuous-Time ΣΔ Modulators Based on a Relative Time-Base Projection. IEEE Trans. Circuits Syst. I Regul. Pap..

[31] Ortmanns M., Manoli Y., Gerfers F. A Continuous-Time Sigma-Delta Modulator with Reduced Jitter Sensitivity. Proceedings of the 28th European Solid-State Circuits Conference.

[32] Hernandez L., Wiesbauer A., Paton S., Giandomencio A.D. Modelling and Optimization of Low Pass Continuous-Time Sigma Delta Modulators for Clock Jitter Noise Reduction. Proceedings of the 2004 IEEE International Symposium on Circuits and Systems (ISCAS).

[33] Ren J., Gregori S. Stacked-Ring Oscillator with Reduced Phase Noise. Proceedings of the 2008 Canadian Conference on Electrical and Computer Engineering.

[34] Wismar U., Wisland D., Andreani P. A 0.2V 0.44 *μ*W 20 kHz Analog to Digital ΣΔ Modulator with 57 fJ/Conversion FoM. Proceedings of the 2006 Proceedings of the 32nd European Solid-State Circuits Conference.

